# Supplementary material for: Validating the CHARMM36m protein force field with LJ-PME reveals altered hydrogen bonding dynamics under elevated pressures
Source: Commun Chem. 2021 Jun 28;4:99. doi: 10.1038/s42004-021-00537-8 (PMC9814493; doi:10.1038/s42004-021-00537-8)
Supplement: Supplementary file 1 — Supplemental Material [file 42004_2021_537_MOESM1_ESM.pdf]

Supplementary Information

Validating the CHARMM36m Protein Force Field with LJ-PME Reveals Altered Hydrogen Bonding Dynamics under Elevated Pressures

You Xu[1,2,3], Jing Huang[1,2,3] *

1. Key Laboratory of Structural Biology of Zhejiang Province, School of Life Sciences, Westlake University, 18 Shilongshan Road, Hangzhou, Zhejiang 310024, China.

2. Westlake AI Therapeutics Lab, Westlake Laboratory of Life Sciences and Biomedicine, 18 Shilongshan Road, Hangzhou, Zhejiang 310024, China.

3. Institute of Biology, Westlake Institute for Advanced Study, 18 Shilongshan Road, Hangzhou, Zhejiang 310024, China.

*Corresponding author: Jing Huang, huangjing@westlake.edu.cn

Tables

Table S1. Linear regression of average atomic RMSF with respect to the pressure. The slopes presented have been multiplied by 10000.

T (K)	α-helices		β-sheets		loops	
	$10^4\ a$	R^2	$10^4\ a$	R^2	$10^4\ a$	R^2
278	-0.22	0.62	0.08	0.10	0.24	0.37
293	-0.40	0.77	-0.53	0.68	-0.43	0.52
308	-0.22	0.68	-0.05	0.06	0.03	0.01
323	-0.11	0.21	-0.05	0.02	-0.08	0.04

Table S2. Statistics of detected H-bonds in simulations with the average effective occupancy greater than 5% over all temperatures and pressures.

	Direct						Water bridged					
Occupancy (%)	Tot.	>70	>50	>30	>10	>5	Tot.	>70	>50	>30	>10	>5
Backbones	53	37	1	5	6	4	3	1	0	1	1	0
Backbone – side chain	30	4	3	7	16	0	58	1	2	6	25	24
Side chains	17	1	2	4	6	4	44	0	4	3	28	9

Table S3. The number of residue duet that has side chain involved H-bonds additional to backbone H-bonds.

	Direct		Water bridged	
Occupancy (%)	Tot.	>20	Tot.	>20
Backbone – side chain	6	2	7	1
Side chains	7	4	7	5

Figures

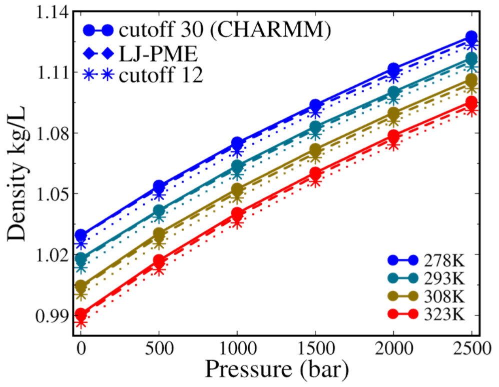

Figure S1. The TIP3P water density with respect to temperature and pressure simulated using different Lennard-Jones computational scheme.

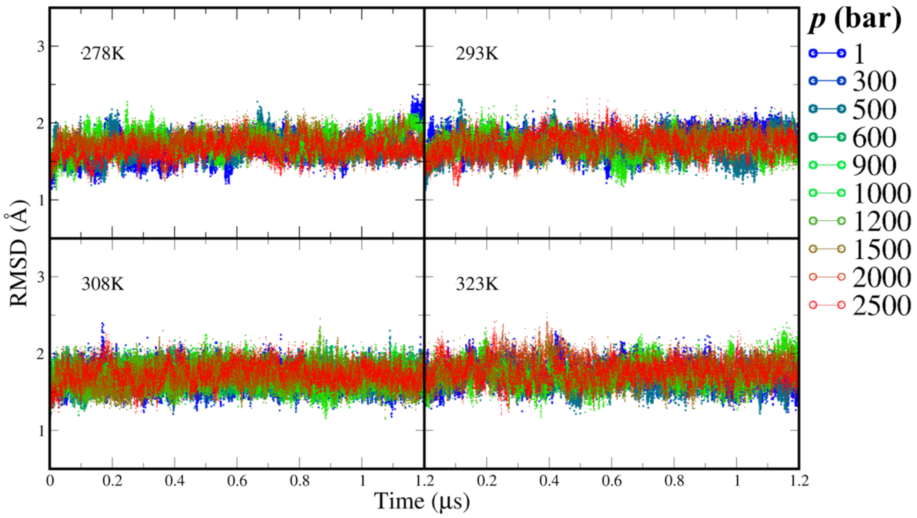

Figure S2. The coordinate RMSD of ubiquitin heavy atoms as the function of the simulation time.

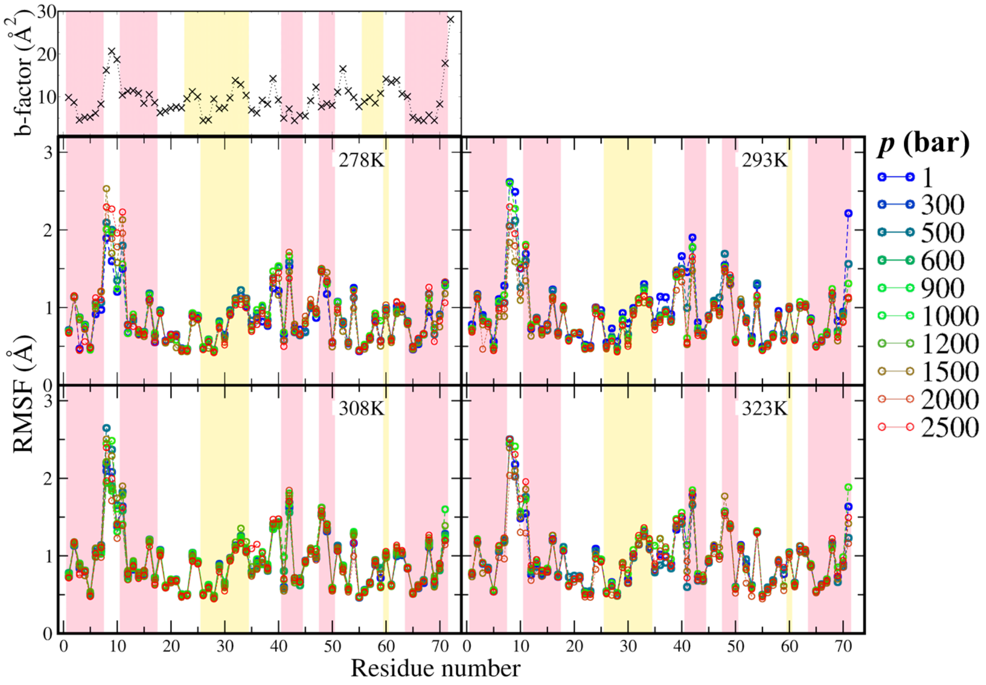

Figure S3. The RMSF of ubiquitin residues 1—71. The designation of secondary structure domains was colored on panel background, as helices in yellow and β-strand in pink. The black cross on the top-left indicates the average b-factor per residue of backbone atoms in the crystal structure 1UBQ.

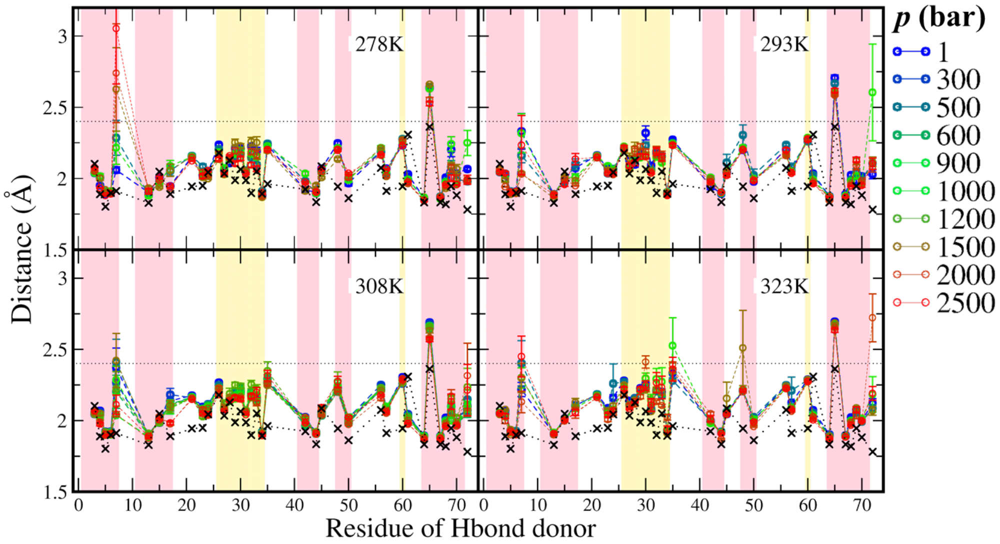

Figure S4. The distance between donor and acceptor of backbone H-bonds in ubiquitin. The hydrogen bonds are indexed by the donor residue. The background is colored as the secondary structure domains, *i.e.* yellow for helices and pink for sheets. The black cross indicates the HN···O distance in the crystal structure 1UBQ (hydrogen atoms were added according to C36m parameters). The error bars show the averaged block standard deviation. The dotted baselines indicate 2.4 Å, under which the interaction is considered to be a strong hydrogen bond.

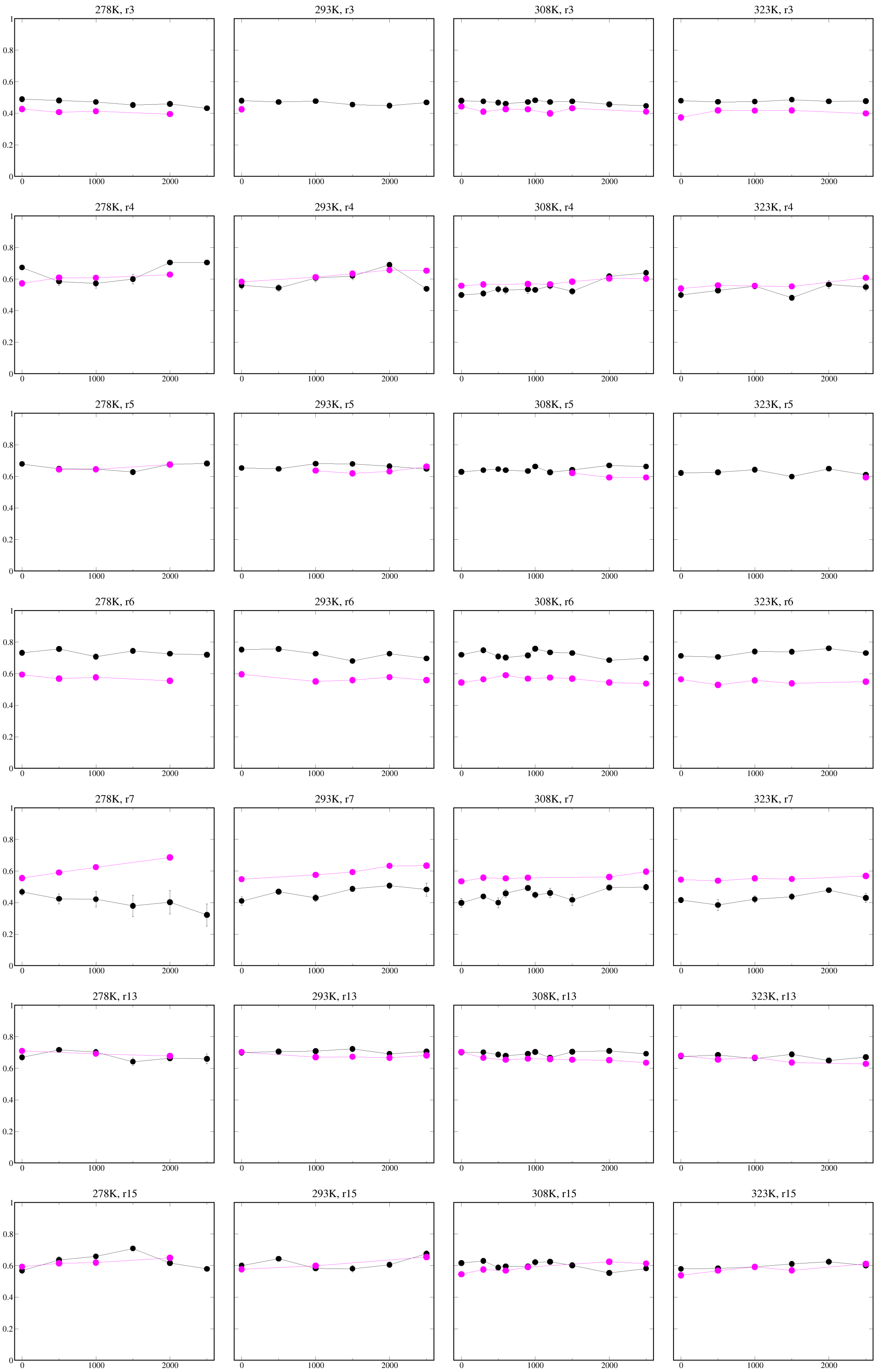
278K, r3
293K, r3
308K, r3
323K, r3
278K, r4
293K, r4
308K, r4
323K, r4
278K, r5
293K, r5
308K, r5
323K, r5
278K, r6
293K, r6
308K, r6
323K, r6
278K, r7
293K, r7
308K, r7
323K, r7
278K, r13
293K, r13
308K, r13
323K, r13
278K, r15
293K, r15
308K, r15
323K, r15
0
0.2
0.4
0.6
0.8
1
0
1000
2000

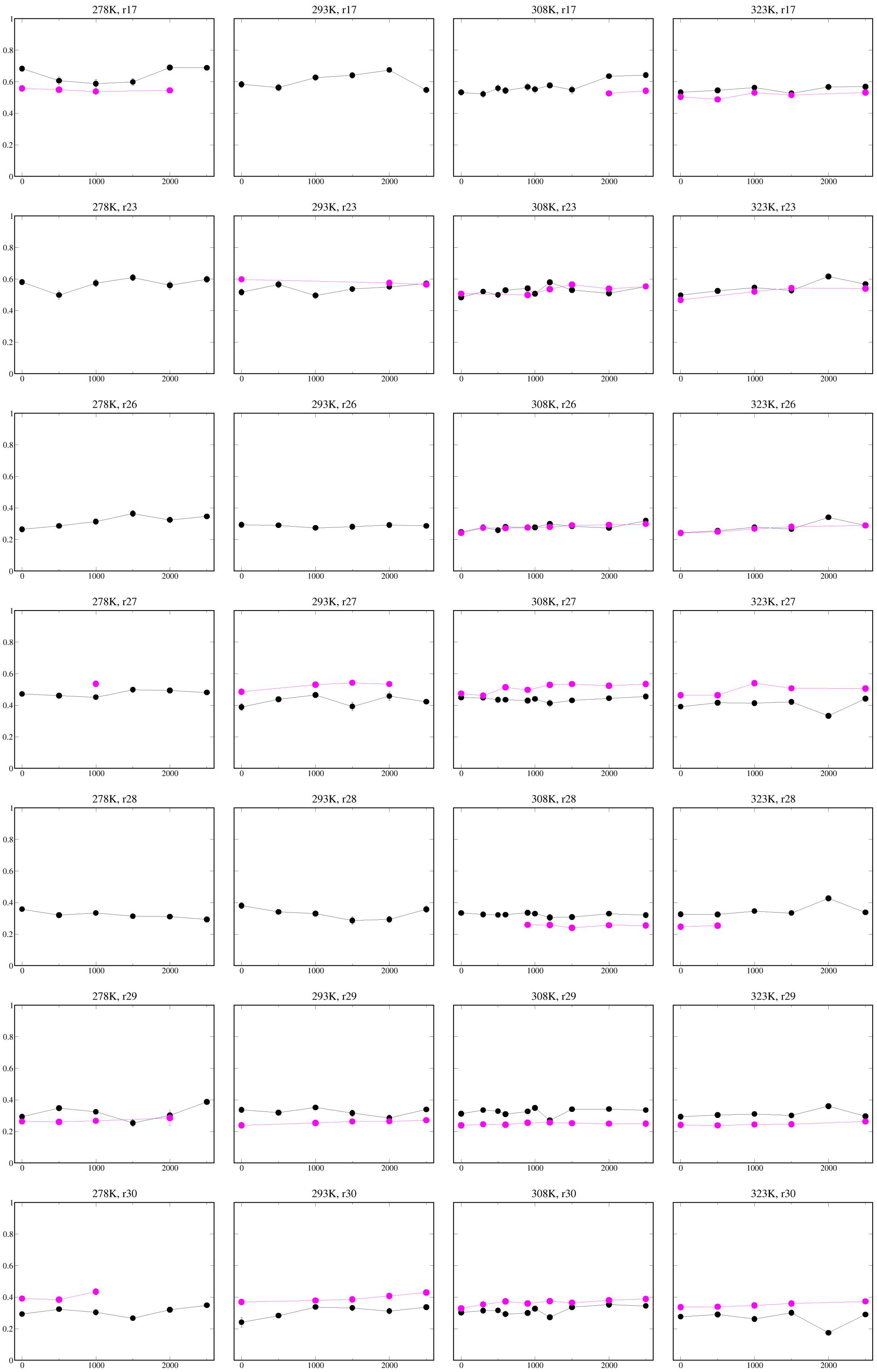
278K, r17
293K, r17
308K, r17
323K, r17
278K, r23
293K, r23
308K, r23
323K, r23
278K, r26
293K, r26
308K, r26
323K, r26
278K, r27
293K, r27
308K, r27
323K, r27
278K, r28
293K, r28
308K, r28
323K, r28
278K, r29
293K, r29
308K, r29
323K, r29
278K, r30
293K, r30
308K, r30
323K, r30
0
0.2
0.4
0.6
0.8
1
0
1000
2000

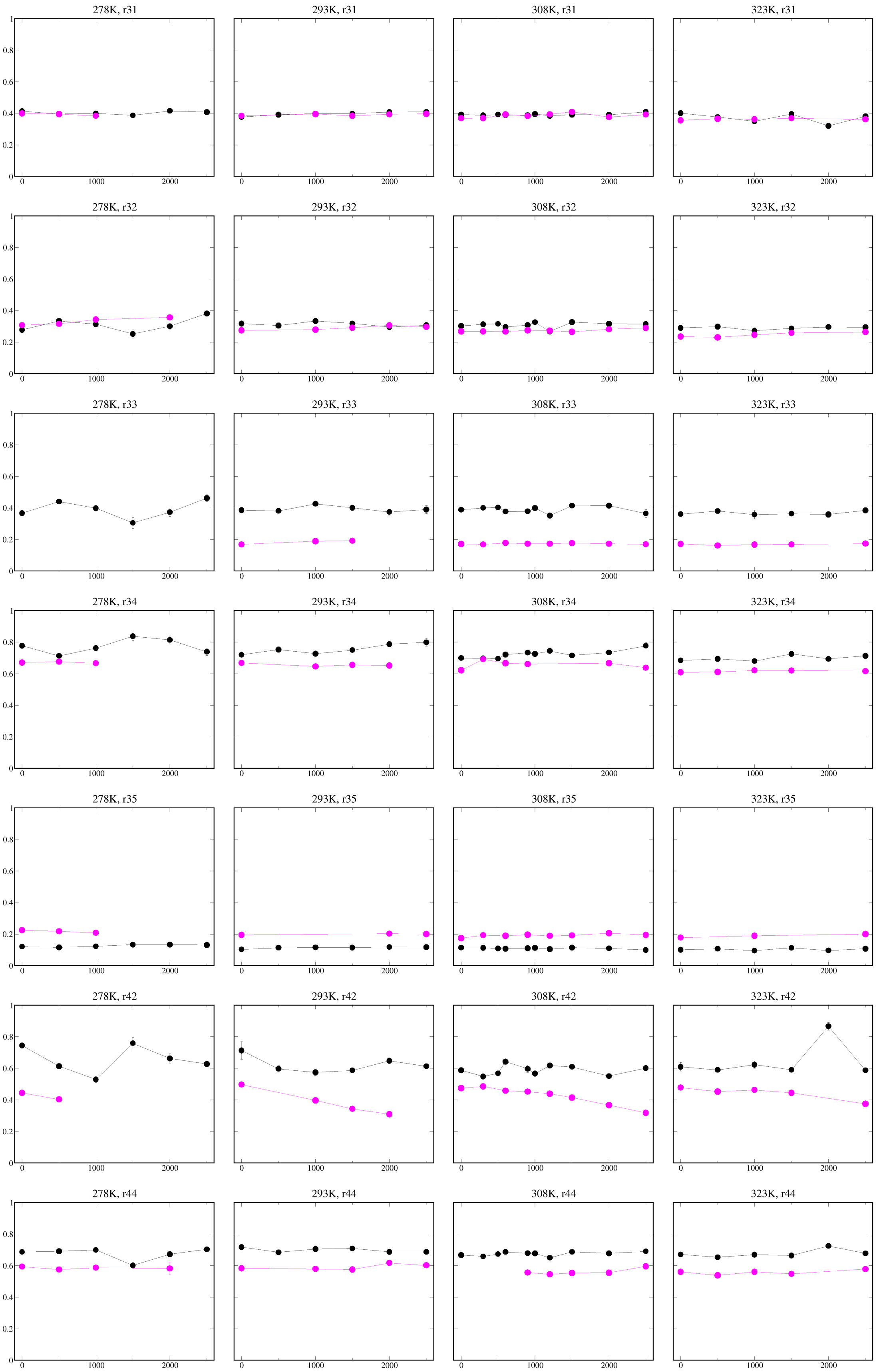
278K, r31
293K, r31
308K, r31
323K, r31
278K, r32
293K, r32
308K, r32
323K, r32
278K, r33
293K, r33
308K, r33
323K, r33
278K, r34
293K, r34
308K, r34
323K, r34
278K, r35
293K, r35
308K, r35
323K, r35
278K, r42
293K, r42
308K, r42
323K, r42
278K, r44
293K, r44
308K, r44
323K, r44
0
0.2
0.4
0.6
0.8
1
0
1000
2000

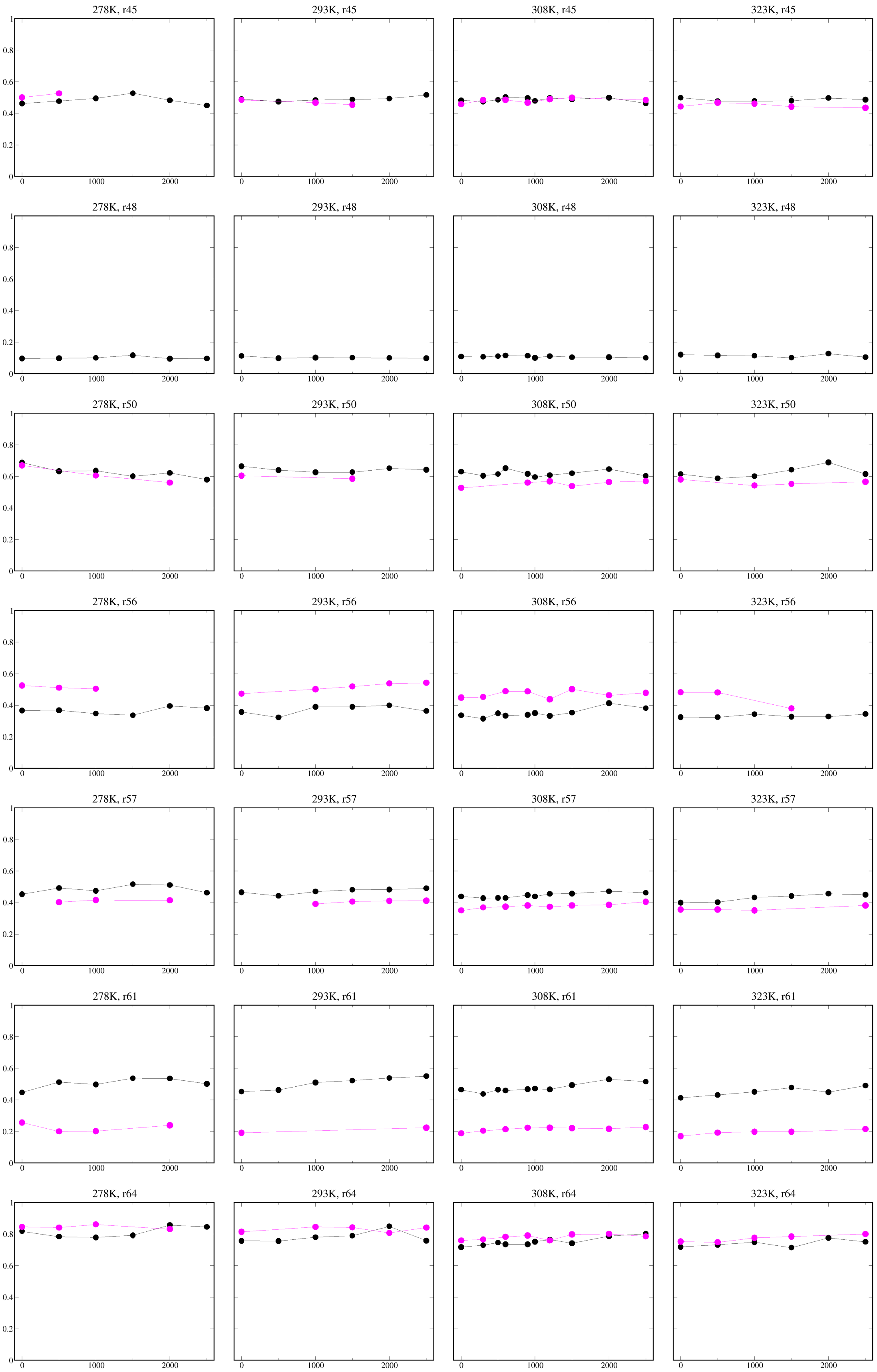
278K, r45
293K, r45
308K, r45
323K, r45
278K, r48
293K, r48
308K, r48
323K, r48
278K, r50
293K, r50
308K, r50
323K, r50
278K, r56
293K, r56
308K, r56
323K, r56
278K, r57
293K, r57
308K, r57
323K, r57
278K, r61
293K, r61
308K, r61
323K, r61
278K, r64
293K, r64
308K, r64
323K, r64
0
0.2
0.4
0.6
0.8
1
0
1000
2000

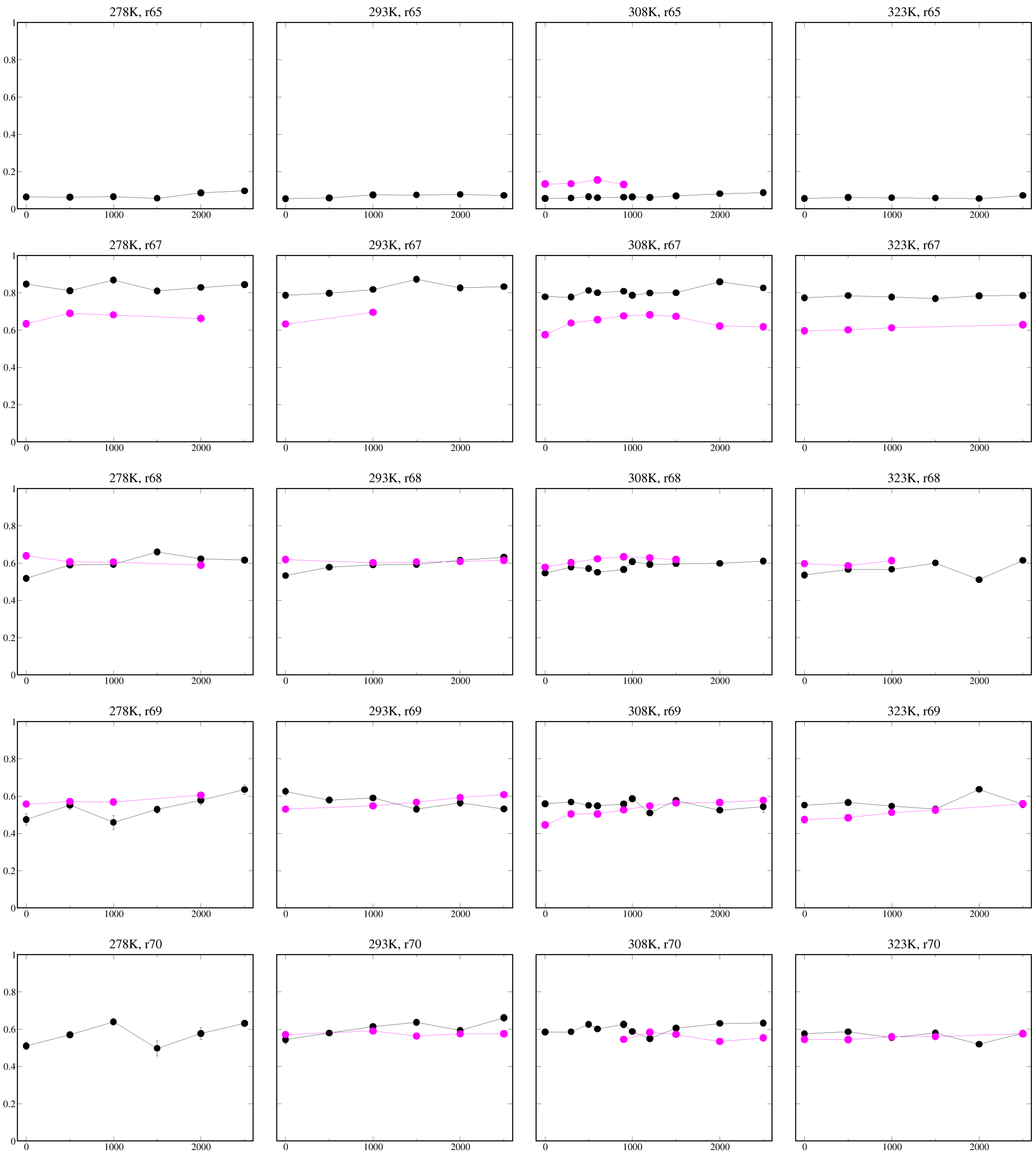

Figure S5. The comparison of residue $^{h3}J_{NC'}$ with respect to pressure. The calculations are in black and experimental $^{h3}J_{NC'}$ data[1] are in magenta. Each row displays four temperature systems for a hydrogen bond with *y*-axis showing the *J*-coupling and *x*-axis showing the pressure. Each panel is titled by temperature and donor residue identifier. The error bars of calculated data show the averaged block standard deviation.

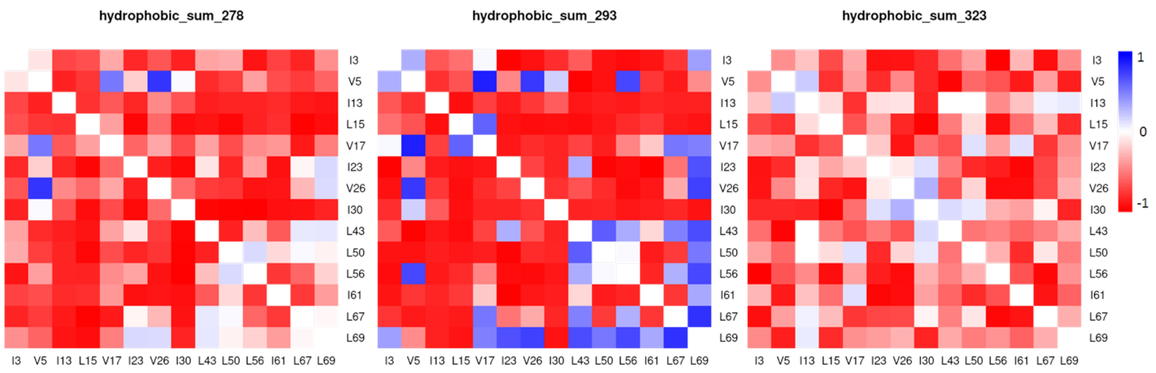

Figure S6. Pearson correlation coefficients between hydrophobic distances and pressure. The pairwise distances were measured between the mass centers of 14 hydrophobic side-chains in the pocket at temperature 278 K (left), 298 K (middle) and 323 K (right). From $\rho = 1$ (blue) to $\rho = -1$ (red), the distances correlate with pressure from positively to negatively.

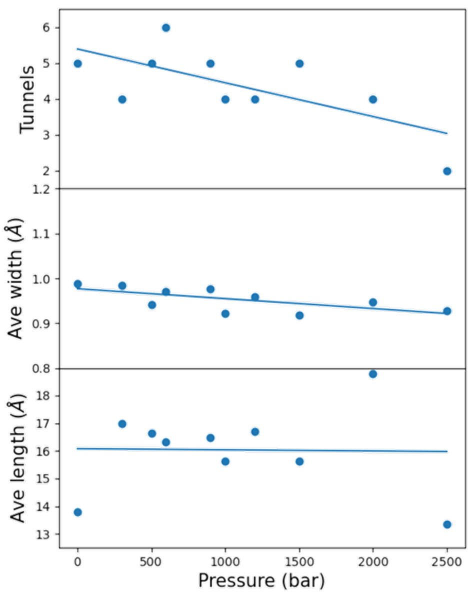

Figure S7. The tunnel parameters for structures at 308 K in response to pressure. The panels of scatter from to bottom show the average tunnel number, average width of tunnel bottleneck, and average length of tunnel. The line shows the linear regression. The analysis was carried out using CAVER 3.0.[2]

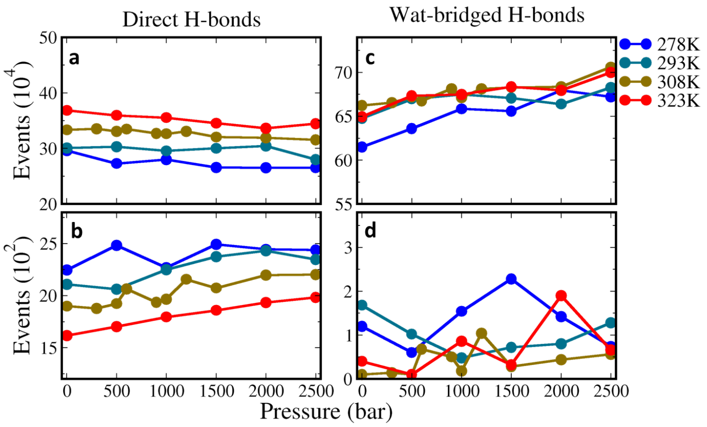

Figure S8. The statistics of event number for hydrogen bonds. The total events of direct H-bond are categorized and presented as lifetime ≤ 6 ns (**a**) and lifetime > 6ns (**b**); the total events of water bridged H-bond are categorized as lifetime ≤ 4 ns (**c**) and lifetime > 4ns (**d**). Note the *y*-axis values of **a** and **c** were scaled by 10^{-4} while the values of **b** and **d** were scaled by 10^{-2}.

Supplementary References

1. Nisius, L.; Grzesiek, S., Key stabilizing elements of protein structure identified through pressure and temperature perturbation of its hydrogen bond network. *Nature chemistry* **2012**, 4, 711-7.
2. Chovancova, E.; Pavelka, A.; Benes, P.; Strnad, O.; Brezovsky, J.; Kozlikova, B.; Gora, A.; Sustr, V.; Klvana, M.; Medek, P.; Biedermannova, L.; Sochor, J.; Damborsky, J., CAVER 3.0: A Tool for the Analysis of Transport Pathways in Dynamic Protein Structures. *PLoS Comp. Biol.* **2012**, 8.
